# Bioenergetic and immunological characterization of cryopreserved peripheral blood mononuclear cells (PBMCs) isolated from blood and buffy coat

**DOI:** 10.3389/fmolb.2025.1716701

**Published:** 2026-01-29

**Authors:** Fabian Dieter, Jendrik Grube, Alice Quentin, Gunter P. Eckert

**Affiliations:** Biomedical Research Center, Institute of Nutritional Sciences, Justus-Liebig-University of Giessen, Giessen, Germany

**Keywords:** bioenergetics, cryopreservation, flow cytometrie, mitochondrial respiration, PBMC (peripheral blood mononuclear cells)

## Abstract

**Background:**

Mitochondrial dysfunction (MD) and inflammaging are hallmarks of non-communicable diseases and play a pivotal role in the ageing process. Determination of mitochondrial function (MF) in Peripheral Blood Mononuclear Cells (PBMCs) represents a minimally invasive method for assessing the pathophysiological state. However, the necessity to measure the cells in a fresh state is a challenge especially in multicenter investigational studies. Therefore, we investigated the mitochondrial and immunological properties of cryopreserved PBMC isolated from whole blood and buffy coats over a storage period of 6 months to establish a procedure to assess MD in frozen samples.

**Methods:**

PBMCs were isolated from whole blood and buffy coats using gradient density centrifugation and cryopreserved with fetal bovine serum and dimethyl sulfoxide as antifreeze agents in liquid nitrogen. To identify potential differences between cryopreserved and freshly isolated cells, we investigated cell count and viability, mitochondrial respiration, adenosine triphosphate production, and citrate synthase activity in PBMCs during 6 months of storing. Cell composition was determined using flow cytometry. The immune status was assessed by determining the cytokines IL-6, IL-10, and TNF-α after phytohemagglutinin stimulation using time-resolved fluorescence (HTRF).

**Results:**

After cryopreservation and storage of PBMCs isolated from whole blood or buffy coats, their bioenergetic function is preserved for at least 6 months: No statistically significant differences in the parameters CS, ATP, and the complexes of the respiratory chain were observed at any measurement time point. However, fluorescence-activated cell sorting analysis reveals that the number of apoptotic cells increased after 1 month of cryopreservation. After 3 months of cryopreservation, phytohemagglutinin-activated cytokines IL-6, IL-10 and TNF-α were significantly increased, indicating a more sensitized immune response of frozen cells.

**Conclusion:**

Cryopreservation of PBMCs has no effect on the measurement of bioenergetic parameters, although increased apoptotic cells are measured. Cryopreserved PBMCs show an increased immunological response, which must be taken into account when interpreting the results.

## Graphical Abstract

**Figure:**
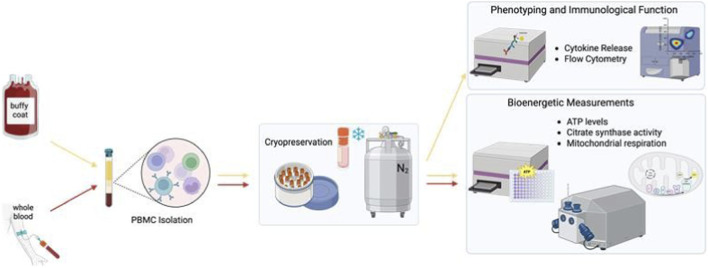

## 1 Introduction

Mitochondria are vital organelles that facilitate the metabolism of most eukaryotic cells, which are reliant on the energy supply from these organelles (Bock and Tait, 2020; Bratic and Trifunovic, 2010). The enzymes of the tricarbon cycle and the respiratory chain, which are located within the inner membrane of mitochondria, are responsible for the production of the majority of energy-rich adenosine triphosphate (ATP) by utilizing energy substrates and oxygen. In addition to their role in energy production, mitochondria play a central role in apoptosis and autophagy. Primary mitochondrial diseases, such as Kearns-Sayre or Leigh syndrome, are associated with mitochondrial dysfunction. Furthermore, the process of ageing and age-related diseases has also been linked to mitochondrial dysfunction (Amorim et al., 2022; Szczesny et al., 2015; Lane et al., 2015; Salminen et al., 2012). However, measuring mitochondrial function in human studies is challenging, especially when biopsies of the affected tissue are not available. This is particularly the case for primary tissues including the brain, where the procurement of tissue samples is not feasible (Hubens et al., 2022). Although muscle biopsy is considered particularly suitable for the assessment of mitochondrial respiration (Pesta and Gnaiger, 2012), it is often limited in its application because it is an invasive procedure. This is due to the fact that skeletal muscle possesses both high energy requirements and a high proportion of mitochondria, thus rendering it a valuable primary tissue sample for the measurement of mitochondrial respiration and ATP synthesis. Alternatively, peripheral blood cells, which have been used for mitochondrial studies in individuals with depression or chronic fatigue syndrome (Gumpp et al., 2021; Vermeule et al., 2010), can be utilized as a primary tissue sample.

We showed in previous studies that PBMCs are a suitable physiological compartment for assessing mitochondrial function (Silaidos et al., 2018; Silaidos et al., 2024; Silaidos et al., 2020; Dieter et al., 2023). Our research also revealed discrepancies between older and younger subjects, as well as gender-related variations (Silaidos et al., 2018; Silaidos et al., 2024). In a recently published protocol we describe that cryopreservation did not affect the mitochondrial parameters: Levels of adenosine triphosphate (ATP), activity of citrate synthase (CS), and of respiratory chain complexes remained constant up to 1 month before and after cryopreservation compared to freshly isolated PBMCs (Dieter et al., 2023).

In other studies, further parameters were determined in PBCMs before and after cryopreservation. The influence on the cell number was determined, the number of antigen-presenting PBMCs and immune response and gene expression were measured (Yang et al., 2016; Martikainen and Roponen, 2020; Lemieux et al., 2016; Ducar et al., 2014; Sadeghi et al., 2013). Other factors influencing the quality of PBMCs are storage temperature, duration and fluctuations of storage conditions (Germann et al., 2013; Angel et al., 2016). Furthermore, transportation (Posevitz-Fejfár et al., 2014) and the used isolation protocol have an impact on PBMC functionality (Corkum et al., 2015; Debey et al., 2004). T cells, as the largest subfraction of PBMCs, are particularly relevant. The analysis of innate immunity and its functions may depend more heavily on the parameters used for freezing and storage (Linder et al., 2024). There are a variety of methods for isolation and cryopreservation of PBMCs. These can be isolated from whole blood or from buffy coats (BC) samples. It is not clear whether both isolates have comparable biological parameters after cryopreservation and storage.

In a first step we examined the role of the cryopreservation period on the mitochondrial parameters of PBMCs isolated from whole blood samples, after one, three, and 6 months of storage at −196 °C using a protocol previously described (Dieter et al., 2023). In the next step, we determined the composition of PBMCs, which were isolated from buffy coats. PBMCs typically consist of lymphocytes, monocytes, natural killer cells (NK cells), and dendritic cells (Godoy-Ramirez et al., 2004; Grievink et al., 2016; Hoffmeister et al., 2003; Kleiveland, 2015). For analysis, flow cytometry was used. In addition, the cytokine response without and after phytohemagglutinin stimulation was investigated, as the cytokine response influences the bioenergetics of PBMCs.

## 2 Methods

### 2.1 Cells

In the first step we examined the role of the cryopreservation period on the mitochondrial parameters of PBMCs, after one, three, and 6 months of storage at −196 °C. Therefore, PBMCs were isolated from whole blood samples of volunteers. The study included four healthy male and four healthy female volunteers aged between 26 and 29. The Ethics Committee of the Justus- Liebig- University Giessen, Germany, approved the study design (reference no. AZ 140/17), which was performed in agreement with the Declaration of Helsinki (Version Fortaleza 2012). After being informed about the test procedure, all test subjects gave their written consent for the experiments to be carried out. Blood samples were collected in the morning at 8 a.m., with the subjects fasting. The test subjects were instructed to refrain from excessive exercise for 48 h prior to the blood sampling. Blood samples used for Peripheral blood mononuclear cells isolation were collected in Lithium-Heparin Monovetten (Sarstedt), and samples for hematology were collected in EDTA-K3 Monovetten (Sarstedt). Exclusion criteria included hemophilia, haematophobia, abnormalities in hematology or intake of anticoagulants. For further investigation of the cell composition PBMCs were isolated from buffy coats (BC) from healthy donors obtained from University Hospital Giessen and Marburg.

### 2.2 Peripheral blood mononuclear cell isolation

To isolate the PBMCs, they were separated from the other components of the blood using density gradient centrifugation and Biocoll Separating Solution (density 1.077 g/mL). The PBMCs were obtained from the residues of blood donations or from whole blood taken from a vein. The blood from the residues of blood donations is referred to as buffy coat in this study. Buffy coats were obtained from whole-blood donations following standard blood bank processing removing plasma and red blood cells. The PBMCs from whole blood and BC did not originate from the same individuals. The blood was mixed 1:1 with Dulbecco’s phosphate-buffered saline (DPBS) (without calcium and magnesium) and carefully layered on top of the Biocoll Separating Solution. Subsequently, the samples were centrifuged for 30 min at 400 g at room temperature with the brakes turned off. After the layers had formed, the PBMCs were carefully removed and transferred to a new 50 mL tube. Isolated PBMCs solution was filled up with DPBS to the 25 mL mark and centrifuged for 10 min at 100 g. The supernatant was removed, and the pellet was resuspended in 25 mL PBS and centrifuged at 100 *g* for 10 min. These washing steps purified the PBMCs from platelets and separation solution. After removal of the supernatant, the pellet was resuspended in RPMI 1640 for cell culture or FBS for cryopreservation.

### 2.3 Cryopreservation of PBMCs

FBS and DMSO were used as cryoprotective media. Both substances were at room temperature (22 °C). The isolated and washed PBMCs were resuspended in 1 mL of FBS and transferred to cryotubes. The cell suspension in the cryotubes was mixed with 1 mL of an FBS-DMSO mixture (80:20) cooled to 4 °C, which was added dropwise to obtain the final concentration of 10% DMSO. For cryopreservation, the cells were adjusted to a density of 10^7^–1.5*10^7^/mL. The cryovials were placed directly into a freezing container precooled to 4 °C so that the cell suspension cooled down in the −80 °C freezer at a controlled rate of 1 °C per minute and stored in the −80 °C freezer for 24 h until they were transferred to liquid nitrogen for longer-term storage.

### 2.4 Thawing of PBMCs

The cryopreserved PBMCs were thawed in a water bath at a temperature of 37 °C until only a pin-sized ice particle was visible. The cryovials were then disinfected and transferred to a sterile workbench. Here, the PBMCs were transferred to 15 mL tubes containing 6 mL RPMI 1640 medium supplemented with 10% FBS, 50 U/mL penicillin and 50 U/mL streptomycin heated to 37 °C. The vials were then rinsed with 2 mL RPMI to transfer any refluxed cells. The cells were centrifuged at 100 *g* for 10 min and the supernatant was removed. The remaining cell pellet was resuspended in 1 mL of fresh RPMI, the cell number was determined and adjusted to the following experiment. To allow the cells to recover from the cryopreservation process and the thawing procedure, they were stored for 24 h in an incubator at 37 °C and 5% CO_2_ saturation.

### 2.5 Cell number and viability

#### 2.5.1 Cell count and viability

The determination of cell number and viability was performed with the TC20™-cell counter (Bio-Rad, Munich, Germany). Trypan blue (Biochrom, Berlin Germany) was used for live dead discrimination.

#### 2.5.2 Flow cytometry

**FIGURE 1:**
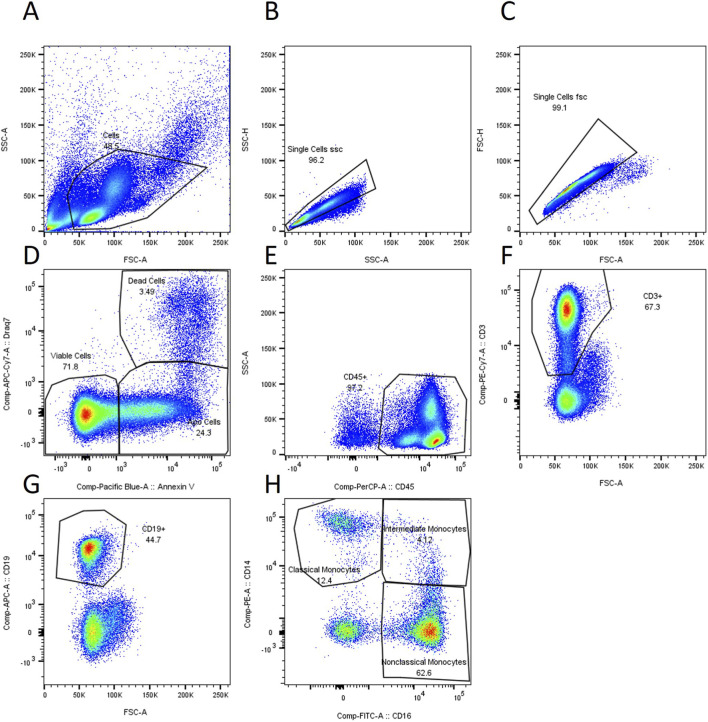
Gating strategy. **(A)** Cells are identified using FSC and SSC. **(B,C)** doublet discrimination **(D)** Dead cells identified as Draq 7 positive events, apoptotic cells identified as Annexin V positive events. **(E)** Identification of CD45 positive cells **(F)** Identification of CD3 positive cells **(G)** Identification of CD19 positive cells **(H)** Identification of CD14 and CD 16 positive cells.

To determine the composition of the PBMCs, they were examined using flow cytometry. These measurements were performed on freshly isolated PBMCs immediately after isolation and on cryopreserved PBMCs immediately after thawing. 10^6^ PBMCs in Hanks’ buffer salt solution were stained with CD 1c, CD 3, CD 14, CD 16, CD 16b, CD 19, CD 45, CD 56, Annexin V and Draq 7 (BioLedgend, California, United States, BD biosciences, California, United States). PBMCs were incubated with the dyes on ice for 30 min. Then, the cells were washed and subjected to flow cytometry analysis (BD FACS Canto II, BD biosciences, California, United States). The gating strategy is presented in Figure 1.

### 2.6 Determination of ATP levels in PBMCs

To determine the ATP levels in PBMCs, they were resuspended after the cryopreservation process in 1 mL of RPMI medium substituted with 10% FBS, Penicilin 50 U/mL Streptomycin 50 U/mL, counted and seeded at a density of 100,000 cells per well in a 96 well plate, this plate was incubated for 24 h in humidified atmosphere supplemented with 5% CO_2_ at 37 °C. This incubation was carried out for freshly isolated cells as well as for cryopreserved cells. ATP concentrations were determined using the ATPlite Luminescence Assay System (Perkin Elmer, Rodgau-Jügesheim, Germany). A luminescence signal is used to determine the ATP level; the emitted luminescence is used to draw conclusions about the ATP content. The signal was analyzed using a ClarioStar plate reader (BMG Labtech, Ortenberg, Germany). To measure the plates, they were taken out of the incubator 15 min before the addition of the reagents to cool them down to room temperature. The cells were lysed for 5 min to release ATP and then mixed with the detection reagent and incubated in the dark for 40 min until they were measured.

### 2.7 High resolution respirometry in permeabilized PBMCs

Mitochondrial respiration was investigated using the Oxygraph-2k system (Oroboros Instruments, Innsbruck, Austria). Two AgCl_2_ electrodes are placed in the independent chambers of the system. The oxygen concentration was determined via the change in resistance using the electrodes over time in the chambers. Substrates and inhibitors of the individual complexes of the mitochondrial respiratory chain are added in a targeted manner in order to measure the respiration of the complexes. This allowed a statement to be made about the functionality of the complexes and the integrity of the respiratory chain in the isolated PBMCs. Prior to the measurement, 2.4 mL MIR05 (37 °C) was added to each chamber and equilibrated for approx. 30 min. For the measurement on cells, the MIR05 was completely removed and replaced by 2.4 mL of cell suspension [8 × 10^6^ cells/mL MIR05] and equilibrated (basal respiration), so that in the end there were 1.6 × 10^7^ cells in each chamber. After stabilization of the basal respiration of the cells, the cells were permeabilized by the addition of digitonin [5 μg/mL] to make the membrane permeable for the substrates and inhibitors. This was followed by the addition of complex I substrates glutamate [10 mM] and malate [2 mM] (leak respiration without ADP, leak (P/M)). The subsequent addition of ADP [2 mM] enabled the detection of complex I activity (CI). The capacity of oxidative phosphorylation (OXPHOS) meaning the total respiration of complexes I and II, was determined by the addition of succinate, which is a substrate of complex II.The addition of FCCP to saturation ([0.5 μM] steps) led to decoupling of the respiratory chain from substrate addition, degradation of the MMP and restoration of the proton gradient during full respiration of the respiratory chain by complex I to complex IV. Thus, the maximum activity of the electron transfer system (ETS) was detected. Subsequently, rotenone [0.5 μM], a complex I inhibitor, was added to differentiate the respiration of complex I from that of complex II. At this stage, the uncoupled complex II activity was detected. Complex III was subsequently inhibited by antimycin A [2.5 μM] and thus enabled the measurement of residual oxygen consumption (ROX), oxygen consumption without the involvement of mitochondrial respiration. Finally, cytochrome c oxidase (complex IV) activity was measured by the administration of tetramethylphenylenediamine (TMPD) [1 mM], an artificial complex IV substrate, and ascorbate [4 mM] to maintain TMPD in a reduced state. The addition of sodium azide [>100 mM] inhibited the entire respiratory chain. The oxygen consumption measured thereafter had to be subtracted from complex IV respiration, as these values no longer originated from the activity of the respiratory chain. Furthermore, ROX was subtracted from all respiratory activities.

### 2.8 Citrate synthase activity in PBMCs

The determination of citrate synthase activity was used in PBMCs as a measure of mitochondrial density and served to normalize mitochondrial respiration by the Oxygraph-2k. Before measuring respiration in the O2k oxygraph, a sample was taken from the cell suspension to determine citrate synthase activity. The method worked on the principle of the irreversible conversion of DTNB to TNB by the enzyme citrate synthase. TNB was determined in the plate reader at a wavelength of 412 nm. The citrate synthase activity was determined from the increase in absorbance per time. The determination was carried out as described by us in this paper (Dieter et al., 2023).

### 2.9 Determination of cytokines

Interleukins and TNF α were determined using homogeneous time-resolved fluorescence (HTRF) before and after stimulation of PBMCs with phytohemagglutinin (PHA). To obtain the samples, 100,000 cells per well were sown on plates and stored in an incubator in humidified atmosphere supplemented with 5% CO_2_ at 37 °C for 24 h. This was carried out in the same way for both cryopreserved and freshly isolated cells. PHA [5 μg/mL] was used to activate the immune function. PHA was added to the cells for 24 h, after which samples were taken from the supernatant. Kits from Revvity (Rodgau-Jügesheim, Germany, 62HTNFAPEG, 62HIL6V2PEG, 62HIL10PEG) were used for the measurement. The measurements were performed according to the manufacturer’s protocol.

### 2.10 Chemicals

The chemicals used for this research were purchased from either Merck, Sigma Aldrich, Thermo Fisher Scientific, or VWR in the highest purity available.

### 2.11 Statistics

Unless stated otherwise, data are presented as mean ± standard error of the mean (SEM). Statistical analyses were performed using Repeated-measures one-way ANOVA (Dunnett test). (Prism 8.0 GraphPad Software, San Diego, CA, United States). Statistical significance was defined for p values *p < 0.05, **p < 0.01, ***p < 0.001, ****p < 0.0001.

## 3 Results

To investigate the influence of long-term storage of PBMCs at cryogenic temperatures, two studies were conducted. In the first study, a cryopreservation period of 6 months was examined. Mitochondrial parameters in the form of ATP, CS, and the respiratory chain were determined in PBMCs isolated from whole blood of volunteers. Cell viability and count were assessed using Trypan blue in an automated cell counter. In a subsequent study the same parameters were measured in PBMC isolated from buffy coats. Additionally, the composition of the cells was determined using flow cytometry and cytokine levels after PHA-stimulation were investigated.

### 3.1 Effect of cryopreservation on cell recovery

**FIGURE 2:**
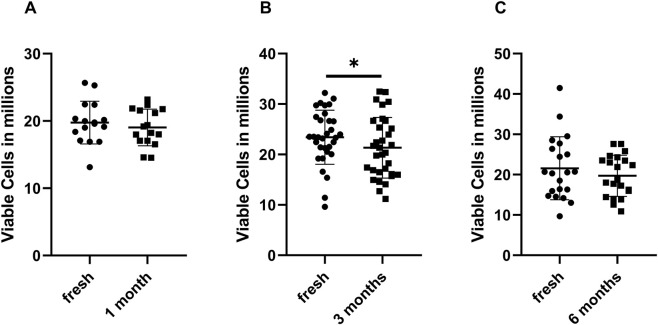
Living cells before and after cryopreservation. Determination of the number of living cells compared to the number of freshly frozen cells and the cell count at the time of thawing after one **(A)**, three **(B)**, and 6 months **(C)**. Values are given as means ± SD. A paired t-test was used to determine statistical significance. n = 16–31 (*p < 0.05). The PBMCs used for this dataset were isolated from whole blood.

To determine whether the cryopreservation process led to a reduction in living cells, each sample was analyzed twice: first in its fresh state after isolation prior to cryopreservation, and then after thawing. The results are shown in Figure 2. The control at each time point corresponds to the freshly isolated cells from whole blood samples in terms of live cells after thawing. After 3 months of cryopreservation, the recovery rate of the cells was reduced; this was not the case for cells cryopreserved for one and 6 months.

### 3.2 Activity of respiration chain complexes (I-IV) before and after cryopreservation

**FIGURE 3:**
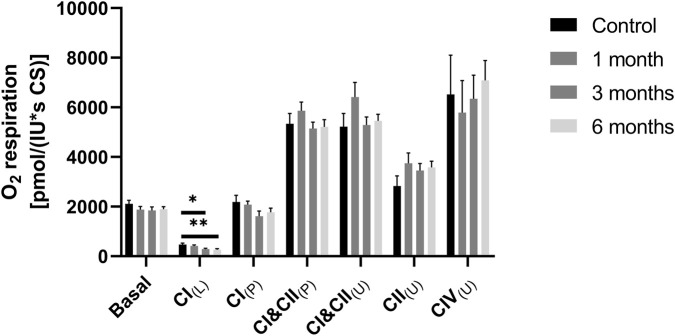
Mitochondrial respiration at different measuring points. PBMCs (8*10^6^/mL) were added to the chambers of the Oxygraph-2k respirometer in order to measure the mitochondrial respiratory chain and its complexes. Figure 3 shows the complexes of the respiratory chain at different measurement times, freshly isolated PBMCs, cryopreserved PBMCs after 1, 3 and 6 months. The basal respiration of the cells, the leak respiration in the presence of glutamate and malate (leak G/M), the respiration of complex I (CI), the OXPHOS (CI&CII), the uncoupled respiratory chain (ETS), complex II after rotenone inhibition (CII_(U)_) and the respiration of complex IV (C IV). The data are given as mean values ±SEM. n = 9. Statistical significance was tested using a Repeated-measures one-way ANOVA (*p < 0.05), (**p < 0.01). The PBMCs used for this dataset were isolated from whole blood.

The respiratory chain plays a central role in energy production. The complexes I, III and IV of the respiration chain build up a proton gradient, which is the driving force for complex V the ATPase, which produces ATP. Oxygen consumption and concentration were measured to determine its function. With the addition of specific inhibitors and substrates, the individual complexes of the respiratory chain were targeted in order to measure their activity. The investigation established that the basal respiration of PBMCs based on their native substrates remained unaffected by cryopreservation at any time point following thawing. However, LEAK respiration of complex I was found to be affected in PBMCs following 3 months (*p = 0.0211) and 6 months (*p = 0.0011) cryopreservation. The LEAK state was determined in the presence of reducing substrate(s), but in the absence of ADP (see Figure 3). Coupled respiration of complex I (CI(P)), oxidative phosphorylation (CI&CII(P)), electron transfer system (CI&CII(U)) and uncoupled respiration of complex II (CII(U)) remained unaffected at all time points in comparison to freshly isolated cells.

### 3.3 Influence of cryopreservation on ATP-Level and citrate synthase activity

**FIGURE 4:**
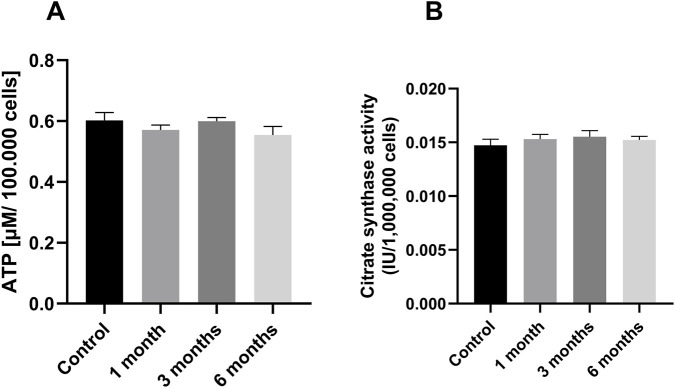
ATP level and Citrate synthase activity: **(A)** The levels of ATP in peripheral blood mononuclear cells (PBMCs) were measured prior to and following a period of cryopreservation ranging from one to 6 months [μM/100,000 cells]. The values obtained are expressed as mean ± SEM, with n = 8. Statistical significance was tested using a Repeated-measures one-way ANOVA. Citrate synthase activity **(B)** Activity of citrate synthase at different measuring points. The citrate synthase activity of PBMCs is shown at the time of collection, after 1 month, after 3 months and after 6 months of cryopreservation. The values obtained are expressed as mean ± SEM, with n = 9. Statistical significance was tested using a Repeated-measures one-way ANOVA. The PBMCs used for this dataset were isolated from whole blood.

The levels of adenosine triphosphate (ATP) and citrate synthase (CS) were measured to determine the activity of the mitochondria. CS has been widely adopted as a marker for mitochondrial mass, which is considered standard practice (Larsen et al., 2012). Mitochondria are the primary producer of ATP; the ATP content provides information about the health and functionality of the mitochondria. The analysis revealed that the application of cryopreservation for a period of 6 months did not result in any observable effect on the concentrations of ATP (see Figure 4A). Furthermore, there were no significant differences between the control group with freshly measured PBMCs and the cryopreserved PBMCs in citrate synthase activity over a period of 6 months (Figure 4B). The result was to be expected, as the activity of the respiratory chain complexes were not or only slightly altered (Figure 3).

### 3.4 Effect of cryopreservation on PBMC subfractions

**FIGURE 5:**
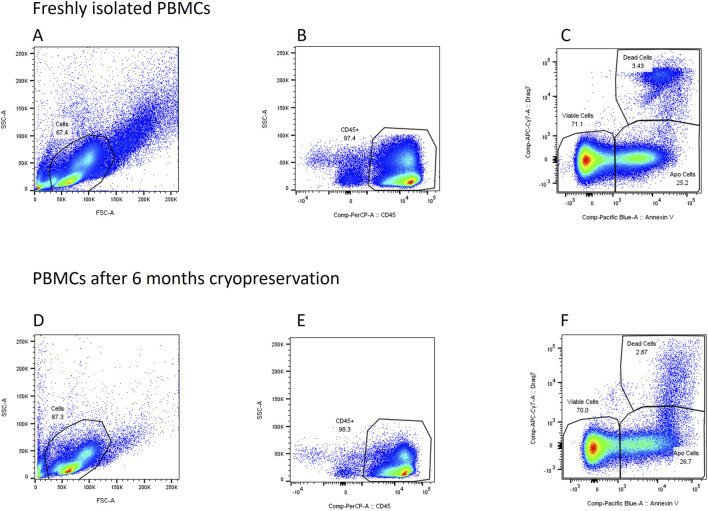
Development over 6 months of cryopreservation. **(A)** Cell identification **(B)** Identification of CD45 positive cells (doublets excluded, not shown) **(C)** Dead cells identified as Draq 7 positive events, apoptotic cells identified as Annexin V positive events. **(A–C)** as freshly isolated PBMCs compared to **(D–F)** after 6 months cryopreservation. The PBMCs used for this dataset were isolated from buffy coats.

**FIGURE 6:**
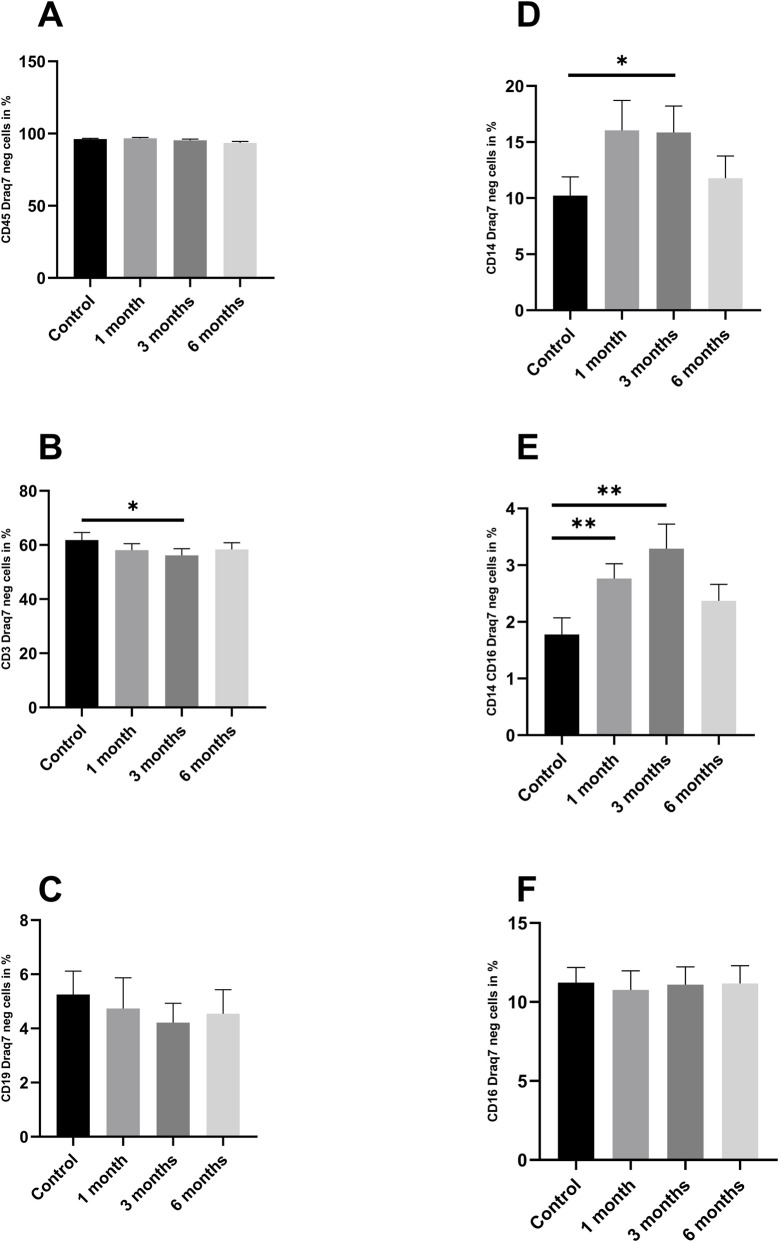
Draq7 negative cells as Fraction of CD45 positive cells: As demonstrated in Figure 1, the figure illustrates various fractions of Draq7 negative cells, including **(A)** CD45 positive cells **(B)**, CD3 positive cells **(C)**, CD19 positive cells **(D)**, CD14 positive cells **(E)**, CD14 and CD16 positive cells **(F)**, and CD14 positive cells. The data are expressed as mean values ±SEM. n = 10. Statistical significance was tested using a Repeated-measures one-way ANOVA (*p < 0.05) (**p < 0.01) (***p < 0.001). The PBMCs used for this dataset were isolated from buffy coats.

**FIGURE 7:**
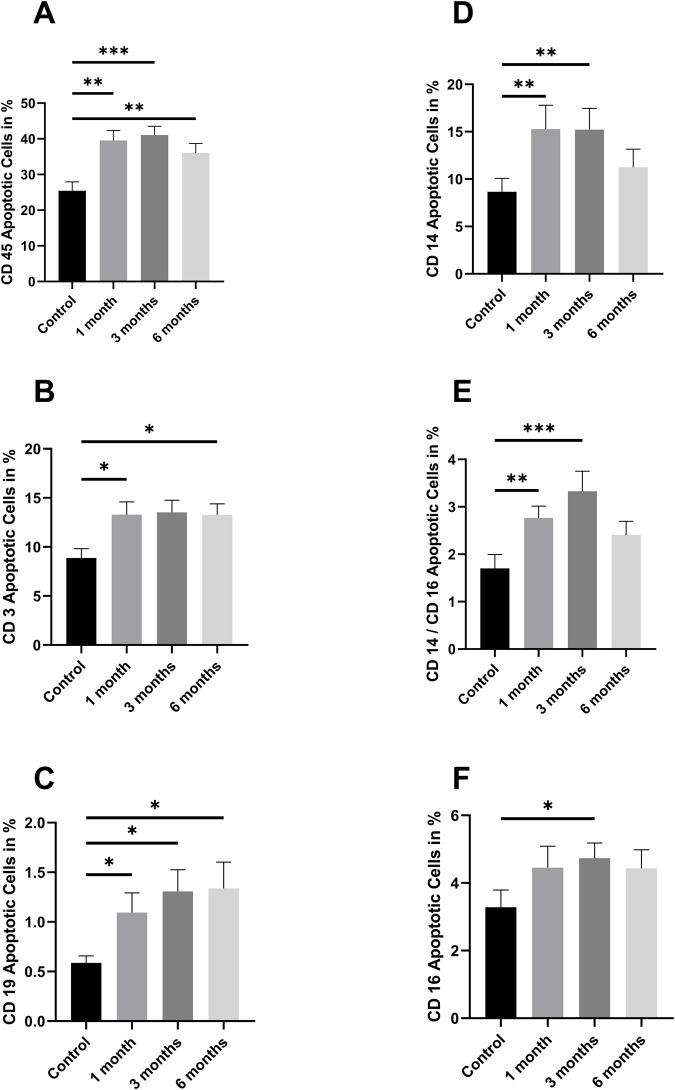
Annexin V positive cells as Fraction of CD45 positive cells: Figure 7 shows different fractions of apoptotic cells **(A)** CD 45 positive cells **(B)** CD3 positive cells **(C)** CD19 positive cells **(D)** CD14 positive cells **(E)** CD 14 and CD 16 positive cells **(F)** CD 14 positive cells. The data are given as mean values ±SEM. n = 10 Statistical significance was tested using a Repeated-measures one-way ANOVA (*p < 0.05) (**p < 0.01) (***p < 0.001). The PBMCs used for this dataset were isolated from buffy coats.

To investigate how cryopreservation affects the different fractions of PBMCs isolated from buffy coats, these were analyzed at different time points using flow cytometry. Figure 5 shows an example of how viability, apoptosis and the number of dead cells changed in PBMCs from a buffy coat over 6 months. To investigate whether cryopreservation affects cell health and how the individual fractions are affected, Annexin V and DRAQ7 were used to perform a differentiated analysis of cell status. Figure 6 represents the Draq7 negative cells and Figure 7 the apoptotic cells.

In the event that cells are Draq7-negative, it can be deduced that their cell membrane is intact. Consequently, these cells are considered to be alive and viable. This is typical for living or early apoptotic cells. Annexin V was used for further differentiation, see Figure 7. The study revealed no statistically significant differences in the proportion of live cells among the CD45, CD19, and CD16 fractions at any of the time points examined (Figures 6A,C,F). However, a divergence was observed in CD3 after 3 months (p = 0.04) (Figure 6B), CD14 after 3 months (p = 0.043) (Figure 6D) and CD14/CD16 after one (p = 0.005) and 3 months (p < 0.001) (Figure 6E).

The proportion of apoptotic cells was increased in all fractions following cryopreservation (Figures 7A–F). For CD45, this increase was observed after 1 month (p = 0.003), 3 months (p < 0.001), and 6 months (p < 0.0047) (Figure 7A). A similar trend was observed for CD3, with a significant increase noted after 1 month (p < 0.0192) and 6 months (p = 0.025). For CD19, the increase was observed after 1 month (p = 0.013), 3 months (p < 0.015), and 6 months (p < 0.0124). For CD14, the results were significant at the 1-month (p = 0.01) and 3-month (p = 0.006) stages. CD14/CD16 positive cells demonstrated significant changes after 1 month (p = 0.006) and 3 months (p < 0.0005). Finally, the CD16 levels were found to be significant after 3 months (p = 0.028).

### 3.5 ATP levels and citrate synthase activity as mitochondrial markers

**FIGURE 8:**
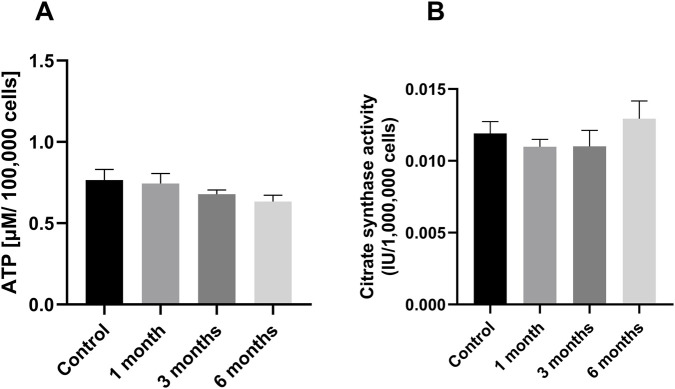
ATP and Citrate synthase activity: Figure 8 shows in **(A)** the level of ATP in peripheral blood mononuclear cells (PBMCs), they were measured prior to and following a period of cryopreservation ranging from one to 6 months [μM/100,000 cells]., **(B)** shows the activity of citrate synthase at different measuring points. The citrate synthase activity of PBMCs is shown at the time of collection, after 1 month, after 3 months and after 6 months of cryopreservation. The data are given as mean values ±SEM. n = 10 Statistical significance was tested using a Repeated-measures one-way ANOVA. The PBMCs used for this dataset were isolated from buffy coats.

The levels of adenosine triphosphate (ATP) and citrate synthase (CS) were measured to determine the activity of the mitochondria in PBMCs isolated from buffy coats. CS has been widely adopted as a marker for mitochondrial mass, which is considered standard practice (Larsen et al., 2012). Mitochondria are the primary producer of ATP; the ATP content provides information about the health and functionality of the mitochondria. The investigation revealed no significant differences. There was also no effect on ATP levels (see Figure 8A) or citrate synthase activity (see Figure 8B) in PBMCs isolated from BC.

### 3.6 Activity of the respiratory chain complexes

**FIGURE 9:**
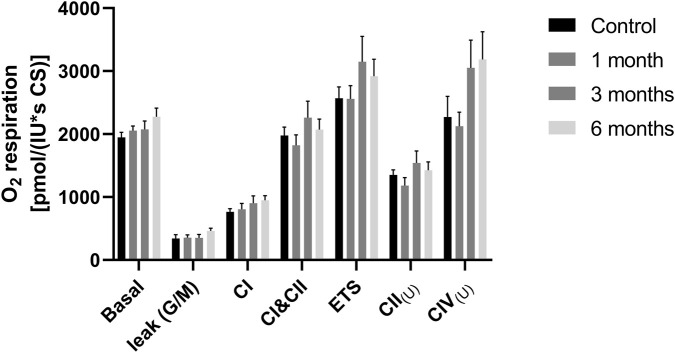
Activity of the respiratory chain complexes: Figure 9 shows the complexes of the respiratory chain at different measurement times, freshly isolated PBMCs from buffy coats (control), cryopreserved PBMCs after 1, 3 and 6 months. The basal respiration of the cells (basal), the leak respiration in the presence of glutamate and malate (leak G/M), the respiration of complex I (CI), the OXPHOS (CI&CII), the uncoupled respiratory chain (ETS), complex II after rotenone inhibition (CII_(U)_) and the respiration of complex IV (C IV). The data are given as mean values ±SEM. n = 10. Statistical significance was tested using a Repeated-measures one-way ANOVA. The PBMCs used for this dataset were isolated from buffy coats.

The activity of the complexes of the respiratory chain and their oxygen consumption were measured in the presence of different inhibitors and substrates in PBMC isolated from buffy coats. Figure 9 shows the results of these measurements. No significant differences were observed between the various time points of measurement.

### 3.7 Cytokine level

**FIGURE 10:**
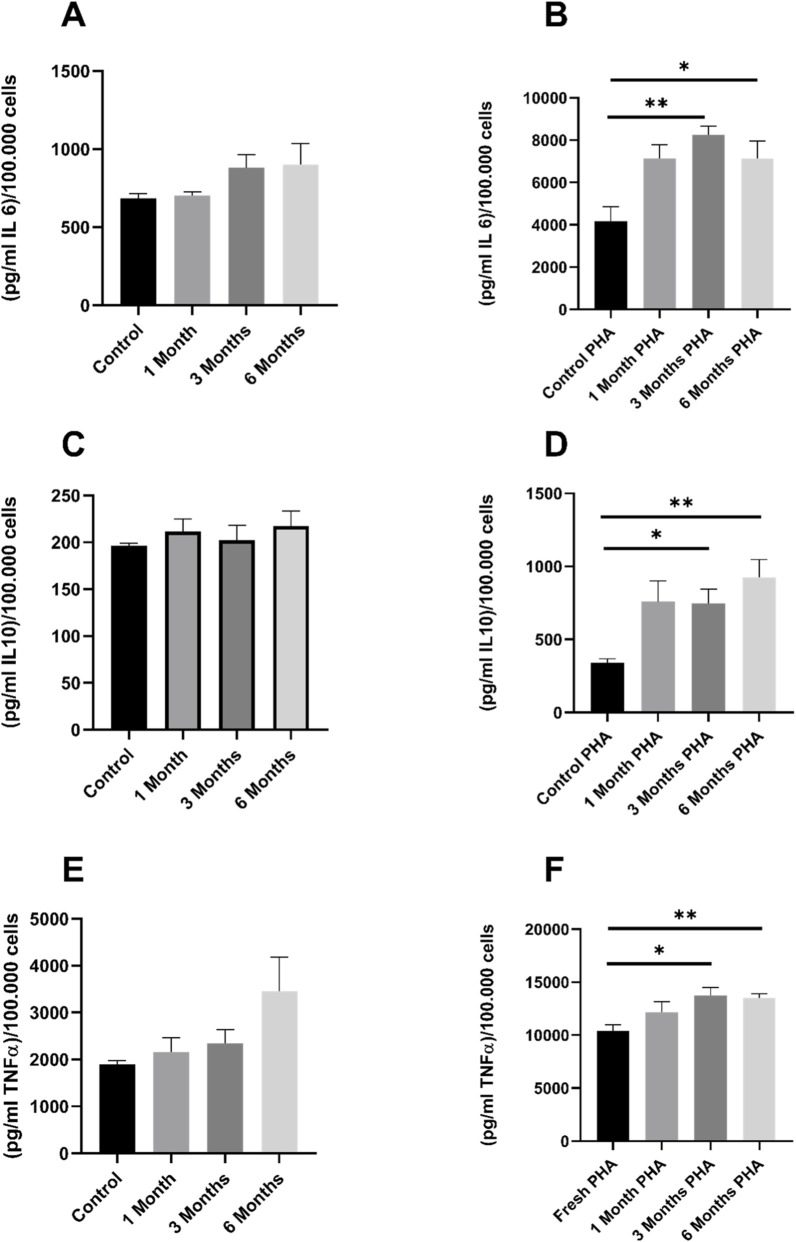
Cytokine levels: Figure 10 shows the cytokines IL 6, IL 10 and TNF-α with and without PHA stimulation at different time points after cryopreservation of PBMCs. The cytokine concentration was measured prior to and following a period of cryopreservation ranging from one to 6 months [(pg/mL)/100,000 cells]. **(A,C,E)** show the respective levels of cytokines without stimulation **(B,D,F)** have been stimulated with PHA. The data are given as mean values ±SEM. n = 10 Statistical significance was tested using a Repeated-measures one-way ANOVA. The PBMCs used for this dataset were isolated from buffy coats.

In order to investigate the effects of cryopreservation on cytokine production, peripheral blood mononuclear cells isolated from buffy coats were examined in the presence or absence of Phytohemagglutinin (PHA) stimulation. Freshly isolated and cryopreserved cells were incubated with and without 5 μg/mL PHA for 24 h. No significant differences were observed in unstimulated PBMCs compared to fresh PBMCs (see Figures 10A,C,E). However, in the groups stimulated with PHA, a significant difference was observed between the cryopreserved cells and the freshly isolated PBMCs after 3 months of cryopreservation. This observation pertains to IL 6, IL 10, and TNF-α. As illustrated in Figure 10B, the levels of IL-6 in PBMCs following PHA stimulation exhibited a statistically significant difference after 3 months (p = 0.002) and 6 months (p = 0.024). Similarly, Figure 10D illustrates the significant difference in IL 10 in PBMCs with PHA stimulation after 3 months (p = 0.013) and 6 months (p = 0.003). Finally, Figure 10F shows the results for TNF-α levels in PBMCs with PHA stimulation, with the relevant p values of 0.015 and 0.009 for the 3-month and 6-month time points, respectively.

### 3.8 Platelet contamination

When isolating PBMCs, it is important to ensure that no residues of the separation solution enter the sample, but other cell compartments can also interfere with measurements. Therefore, washing steps were implemented to keep this contamination as low as possible. It is well established that platelets are significant contaminants of PBMC samples, and their effect on the measurements should be given due consideration (Rausser et al., 2021).

Approximately 99% of the platelets are removed during the washing process (Table 1). The number of platelets remains approximately constant after freezing and storage (Table 2). It is therefore possible to largely rule out any influence on the measurements shown due to possible platelet contamination.

**TABLE 1:** Platelet count before and after washing steps.

Before washing steps	After washing steps
7,021,000 ± 4,747,136/µL	60,700 ± 38,169/µL (p < 0.001)

**TABLE 2:** Platelet count at different time points.

Time point	Platelet count
Fresh	60,700 ± 38,169/µL
1 month	59,200 ± 38,096/µL
3 months	63,600 ± 45,746/µL
6 months	63,700 ± 40,888/µL

### 3.9 Influence of cryopreservation on N/NS flux control ratio

The N/NS ratio indicates the proportion of complex I-dependent respiration in the total maximum electron flux (when both complexes are active). Depending on the result, conclusions can be drawn about the respective proportions of NADH and succinate in the flux. Tables 3, 4 show the N/NS flux control ratio of the PBMCs.

There was no difference in the N/NS ratio at the different measurement times for either PBMCs isolated from whole blood or PBMCs derived from BC.

**TABLE 3:** N/NS flux control ratio in PBMCs for whole blood.

Time point	N/NS flux ratio
Fresh	0.413 ± 0.099
1 month	0.363 ± 0.091
3 months	0.331 ± 0.095
6 months	0.342 ± 0.084

**TABLE 4:** N/NS flux control ratio in PBMCs for whole blood.

Time point	N/NS flux ratio
Fresh	0.395 ± 0.076
1 month	0.443 ± 0.066
3 months	0.404 ± 0.095
6 months	0.464 ± 0.065

## 4 Discussion

The cryopreservation of PBMCs offers advantages in many areas when conducting and planning studies. Samples can be collected and measured centrally, which increases the quality and comparability of results. This centralized collection enables the analysis of a larger number of samples and the application of standardized procedures, which improves the reproducibility of the results. In addition, cryopreservation enables long-term storage of samples so that they can be used for different research purposes at a later date. This is particularly advantageous in longitudinal studies where samples are required over a longer period of time. The ability to compare PBMCs from different time points or under different conditions can provide valuable insights into immunological responses or disease progression. However, it should be noted that freezing PBMCs can lead to changes in the composition and possibly also in the properties of the frozen cells. These changes can be influenced by various factors, such as the cryoprotective substance used (e.g., DMSO), the freezing and thawing speed and the storage conditions. Therefore, both the functional level of the PBMCs and how the composition can change must be taken into account.

The basic requirement for functional measurements on PBMCs is the viability of the cells after the cryopreservation process, which must meet certain standards as otherwise the cells are no longer able to fulfill their normal functions. In 2000, Weinberg and colleagues analyzed the results of the cryopreservation quality control program performed for the immunological component of the AIDS Clinical Trial Group 360 protocol, showing that preparations with a viability of at least 70% showed consistent proliferative responses and were suitable for functional analyses (Weinberg et al., 2000). The viability and subsequent functionality of PBMCs is determined by a variety of factors, the choice and amount of antifreeze agents such as DMSO and subsequent composition of the cryopreservation agent, the temperature of the cryopreservation agent upon cell contact and more (Nazarpour et al., 2012). We demonstrate that PBMCs isolated from whole blood samples were stable in number and viability. On average, 96.2% of living cells could be recovered after 1 month, 91.1% after 3 months, and 91.4% after 6 months. These values are well above the 70% proposed by Weinberg et al. The aforementioned tests were conducted using a cell counter that did not differentiate the cell fractions of PBMCs. In order to make more detailed observations regarding the composition of PBMCs and to investigate the influence of cryopreservation on the individual cell fractions, a flow cytometry analysis was performed on BC-derived PBMCs. The results demonstrated that cryopreservation of BC-derived PBMCs had no effect on cell survival after a 6-month period (3% dead cells after isolation, 1.92% after 1 month of cryopreservation, 3.15% after 3 months of cryopreservation, and 4.04% after 6 months of cryopreservation). However, it should be noted that cryopreservation led to an increase in CD 14/16-positive Draq7-negative cells. There could be two different reasons for this. On the one hand, cryopreservation could have caused a population shift. The increase in TNF-a and IL 6 could plausibly explain this. However, it could also be a technical artifact, as CD 14 staining is not always stable after freezing and thawing processes. The number of apoptotic cells increased significantly. An increased apoptosis rate was observed in each individual fraction following cryopreservation. The increase was evident after 1 month of storage and maintained its level throughout the subsequent months. It appears that the cryopreservation process itself plays a greater role than the preservation time. There is a uniform increase, with no specific cell fraction standing out. There are no differences in dead cells. Through this uniform increase, it can be concluded that no cell fraction is overrepresented or underrepresented.

Once a protocol has been established that meets the requirements for viability and the composition, the next question is whether functions of the preserved cells are affected by cryopreservation. Bioenergetic measurements show an inconsistent picture regarding the functionality of PBMCs before and after cryopreservation. Following the process of cryopreservation, a decline in cellular bioenergetics was observed after a period of 4 weeks. After 1 month of storage, mitochondrial function was impaired, as indicated by a decline in basal respiration, ATP production, maximal respiration, reserve capacity, and coupling efficiency (Keane et al., 2015). Similar findings were also observed by Jones et al. Here, some measures of oxidative phosphorylation in particular, but also glycolysis, were affected (Jones et al., 2015). ATP levels were reduced by cryopreservation when cells were subjected to experiments immediately after thawing, but ATP concentrations returned to normal levels when cells were allowed to recover for 48 h (Sadeghi et al., 2013). Other studies using cryopreserved PBMCs have not observed differences in the respiratory chain between freshly isolated and cryopreserved PBMCs (Germann et al., 2013; Grievink et al., 2016; Boeck et al., 2018; Boeck et al., 2016). There are many possible reasons for these different results; it is possible that the methods used respond differently to cryopreservation. Also, the method of measuring mitochondrial activity may affect the outcome of studies. For the determination of respiratory chain activity, Seahorse XF showed reduced values, whereas this was not observed for measurements in the Oxygraph-2k. The Seahorse XF assay is fluorescence-based, whereas the oxygraph uses a Clark electrode, making it an electrochemical method. The Seahorse assay uses intact cells, whereas the oxygraph uses permeabilized cells treated with digitonin. However, this is not necessarily due to the method used but may also have other causes. Other reasons could be the type of preservation medium, as differences in viability already have been described (Nazarpour et al., 2012), and it has also been shown that the thawing of the cells and especially the temperatures of the water bath as well as the washing medium play an important role (Hønge et al., 2017). In the course of our experimental inquiries, we were unable to ascertain any statistically significant discrepancies between the groups with respect to the parameters CS, ATP, and the complexes of the respiratory chain. The calculated N/NS ratio also shows no differences between the various measurement times. However, with values around 0.4 on average, it was lower than that described by others for PBMCs, where a value between 0.50 and 0.75 was determined (Timon-Gomez et al., 2024). However, as this was also the case for freshly isolated PBMCs, this lower value cannot be attributed to cryopreservation. The only bioenergetic parameter that was altered was the LEAK respiration of Complex I, which was reduced after cryopreservation of PBMCs from whole blood. This indicates healthier mitochondria. This observation, which seems counterintuitive at first, may be explained by the fact that damaged mitochondria are eliminated by the preservation process. However, the same effect could not be observed in PBMCs from BC. Our results indicate that our method of cryoconservation provides stable bioenergetic mitochondrial function over a storage time of at least 6 months at −196 °C. The bioenergetic measurements were performed after a recovery period of 24 h. This was done to ensure optimal recovery of the PBMCs, but it has the disadvantage of potentially limiting the clinical usefulness. Others have shown that PBMCs can be measured in the O2k oxygraph without a 24-h recovery period and with no change in the measured values (Payne et al., 2025).

Regarding the effect of cryopreservation on the immune response and cytokine release, different results have also been observed. While in some studies no differences were observed between cryopreserved and fresh cells, in others an increase or decrease in cytokine levels were observed after cryopreservation: Cryopreserved PBMCs produced fewer cytokines in response to stimulation than fresh cells, but the response profiles were comparable (Martikainen and Roponen, 2020). PBMCs cryopreserved in whole blood experienced no functional impairment in intracellular cytokine production (Braudeau et al., 2021). Jones et al. observed a change in OXPHOS and glycolysis, but no change in cytokine release (Jones et al., 2015). Increases in cytokines have also been described (Venkataraman, 1997; Ande et al., 2019). In these studies, cytokine release was mediated by LPS. A mixed picture was observed after PHA activation, with increased cytokine release in cryopreserved PBMCs, while no differences were detected in non-activated cells. As bioenergetics measurements were also performed in non-activated cells and no differences were found between the groups, these results seem to be consistent. The ratio between TNF-α and IL-10 is often used to assess macrophage activation. After cryopreservation, there was a time-independent increase in IL-10 and TNF-α, with IL-10 showing a relatively stronger increase. The increased IL-10 release in frozen PBMCs artificially shifts the TNF-α/IL-10 ratio toward an anti-inflammatory signature. This can lead to misinterpretations of macrophage activation, distortions in immune profiles, and limitations in the comparability between fresh and frozen samples. Cryopreservation is therefore an active biological factor that alters the cytokine profile and must be taken into account in the analysis. One possible reason for the observed results could be granulocytes, which are very susceptible to the effects of cryopreservation and therefore do not survive it (Blanter et al., 2021). Granulocytes are separated during density gradient centrifugation, but it cannot be ruled out that there were residues in the fresh sample, leading to a different cytokine response between fresh and cryopreserved cells. This can also be seen in Figure 5, where granulocytes are greatly reduced after 6 months of cryopreservation, while lymphocytes and monocytes remain intact. The freshly isolated sample contains more granulocytes.

Cryopreservation has been shown to influence cytokine release, but this change appears to be stable over a period of 1–6 months; however, a longer preservation time does not appear to result in any further increase. Cryopreservation may have caused changes in the PBMCs that made them more susceptible to PHA stimulation. Changes in the cell membrane and changes in signal transduction could lead to increased cytokine release. Another factor leading to increased cytokine release is apoptotic signals in PBMCs. Since the apoptosis rate was increased after cryopreservation, this is another possible reason for the higher values. However, cell damage can also lead to an increased release of cytokines under PHA stimulation. Studies using stimulants should take this into account.

The limitations of this study include the limited comparability of PBMCs from whole blood and PBMCs from BC. In order to achieve the necessary cell count to perform all experiments, BC must be used; however, the results from PBMCs from whole blood are still important findings, as whole blood is used in clinical practice. Nevertheless, bioenergetic measurements showed the same results regardless of the origin of the PBMCs. Another limiting factor is the study population used. In order to obtain a more complete picture of the effects of cryopreservation, a wider range of donors is essential, as the whole blood donors were only young, healthy subjects.

## 5 Conclusion

Our results show that cryopreserving PBMCs is a reliable method, as mitochondrial bioenergetics are not impaired. However, the exact cell composition should be checked by flow cytometry, since different immune cell subtypes have distinct properties. Even though cryopreserved samples are overall comparable—because the subpopulation effects are similar—it is still important to isolate and study individual subtypes separately. This underlines the need to consider how cryopreservation might influence immune cell responses in clinical studies, even if it appears to be less critical for bioenergetic analyses. In any comparison, the protocols used for cell isolation, freezing, thawing, and culture should always be taken into account.

## Data Availability

The raw data supporting the conclusions of this article will be made available by the authors, without undue reservation.
