# Supplementary material for: Bioenergetic and immunological characterization of cryopreserved peripheral blood mononuclear cells (PBMCs) isolated from blood and buffy coat
Source: Front Mol Biosci. 2026 Jan 29;12:1716701. doi: 10.3389/fmolb.2025.1716701 (PMC12894023; doi:10.3389/fmolb.2025.1716701)
Supplement: Supplementary file 1 [file Table1.docx]

Supplementary table 1

| Antibody | Fluorochrome | Supplier | Catalogue Number | Vol. per Test [µL] |
| --- | --- | --- | --- | --- |
| Annexin V | Pacific Blue | BioLegend | 640918 | 5 |
| CD1c | BV510 | BioLegend | 331534 | 5 |
| CD56 | BV510 | BD Biosciences | 563041 | 5 |
| CD16 | FITC | Invitrogen | MA1-19611 | 5 |
| CD14 | PE | BioLegend | 325606 | 5 |
| CD45 | PerCP | BD Biosciences | 345809 | 20 |
| CD3 | PE/Cy7 | BioLegend | 344816 | 5 |
| CD19 | APC | BioLegend | 363006 | 5 |
| DRAQ7 | APC/Cy7 | BioLegend | 424001 | 3 |

The following tests were performed to establish the panel. Unstained, single stains, cocktail stains, full stains, and FMOs were measured. Compensation was performed for all antibody conjugates with beads.
