# Supplementary material for: Bioenergetic and immunological characterization of cryopreserved peripheral blood mononuclear cells (PBMCs) isolated from blood and buffy coat
Source: Front Mol Biosci. 2026 Jan 29;12:1716701. doi: 10.3389/fmolb.2025.1716701 (PMC12894023; doi:10.3389/fmolb.2025.1716701)
Supplement: Supplementary file 2 [file Image1.pdf]

A

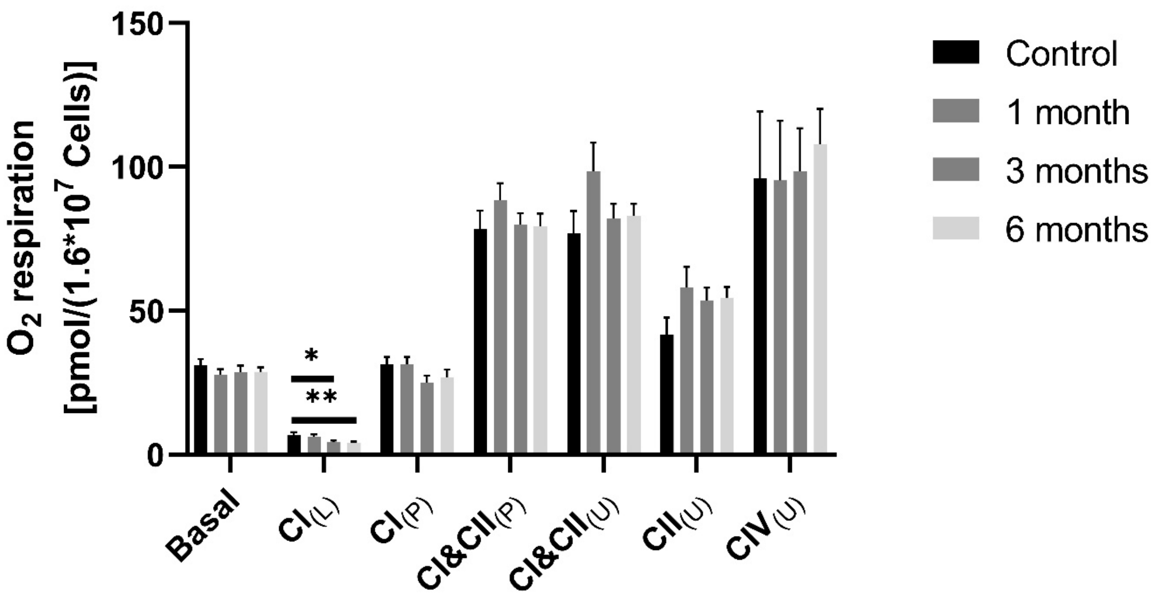

B

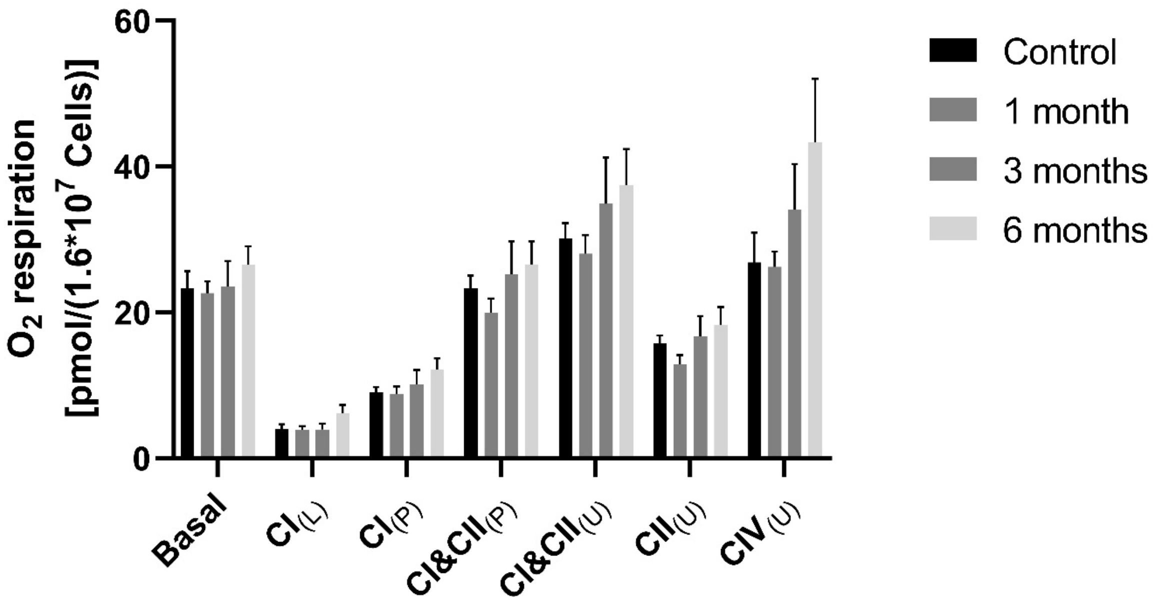

Supplementary Figure 1 Activity of the respiratory chain complexes: Supplementary Figure 1 shows the complexes of the respiratory chain at different measurement times, **A** freshly isolated PBMCs from whole blood (control), cryopreserved PBMCs after 1, 3 and 6 months. **B** freshly isolated PBMCs from buffy coats (control), cryopreserved PBMCs after 1, 3 and 6 months. The basal respiration of the cells (basal), the leak respiration in the presence of glutamate and malate (leak G/M), the respiration of complex I (CI), the OXPHOS (CI&CII), the uncoupled respiratory chain (ETS), complex II after rotenone inhibition ($CII_{(U)}$) and the respiration of complex IV (C IV). The data are given as mean values ± SEM. n = 10. Statistical significance was tested using a Repeated-measures one-way ANOVA.
